# From Genotype to Functional Risk: A Multi-Omic Approach to Predicting Thiopurine and Methotrexate Co-Therapy-Induced Liver Injury

**DOI:** 10.3390/ph19050733

**Published:** 2026-05-06

**Authors:** Dénes Molnár, Elizabeth Reznik, Pálma Porrogi

**Affiliations:** 1 Centre for Translational Medicine, Semmelweis University, 1085 Budapest, Hungary; molnarjozsefdenes@gmail.com; 2 NYU Langone Hospital, New York, NY 11220, USA; elizabeth.reznik@nyulangone.org; 3 Faculty of Health and Sport Sciences, Széchenyi István University, 9026 Győr, Hungary

**Keywords:** thiopurine, methotrexate, phenoconversion, multi-omics, drug-induced liver injury, genotype-phenotype gap, acute lymphoblastic leukemia

## Abstract

The combination of thiopurine and methotrexate (MTX) is a standard co-therapy regimen for acute lymphoblastic leukemia (ALL). Despite its efficacy, this regimen is constrained by a narrow therapeutic window and considerable inter-individual variability, which heightens the risk of drug-induced liver injury (DILI). MTX-induced metabolic strain further destabilizes cytokine-sensitive thiopurine detoxification pathways during systemic inflammation. Conventional pharmacogenetic (PGx) testing for *TPMT* and *NUDT15* variants is effective in predicting myelosuppression, but often fails to detect hepatotoxicity as an adverse effect, suggesting a clinically significant genotype-phenotype difference. This review examines the molecular determinants of DILI, emphasizing the role of secondary metabolic pathways and transporter dynamics as key modulators of risk. The study describes cytokine-mediated (IL-6, TNF-α) transcriptional suppression of cytochrome P450 enzymes and hepatic transporters (*SLCO1B1*, *ABCC2/4*) not merely as secondary modulators, but as the primary determinants of localized, tissue-specific drug exposure through disrupted nuclear receptor signaling (PXR, CAR, HNF4α). This mechanism promotes functional phenoconversion and toxic molecular shunting, leading to increased intrahepatic drug exposure. It synthesizes the current knowledge on the metabolism of thiopurine and MTX, focusing on the genetic and non-genetic factors influencing toxicity and their interactions. The review also critically evaluates the limitations of static PGx-guided dosing. It highlights the need for comprehensive, real-time risk assessment that integrates gene-environment interactions, multi-omics data, and clinical monitoring to improve precision therapy for ALL. This approach combines extended PGx profiling, transcriptomic monitoring, and clinical biomarker assessment to provide a transformative strategy for precision drug delivery.

## 1. Introduction

Thiopurins such as azathioprine (AZA), 6-mercaptopurine (6-MP), and 6-thioguanine (6-TGP) are immunosuppressants and antimetabolic drugs used to treat autoimmune, cancer, and inflammatory disorders: inflammatory bowel disease (IBD), specifically Crohn’s disease, ulcerative colitis, and acute lymphoblastic leukemia (ALL) [1,2,3]. Methotrexate is an alternative immunomodulator for IBD patients who are intolerant or unresponsive to thiopurines, and it has its own distinct profile of hepatotoxicity. MTX itself is associated with cumulative liver toxicity, including steatosis and fibrosis, especially with long-term use. The combination of 6-MP and methotrexate (MTX) is primarily used to maintain remission. These agents are particularly valuable in patients who are steroid-dependent, enabling corticosteroid-sparing control of disease activity through long-term suppression of aberrant immune responses. Their role has been re-evaluated with the advent of biologic therapies, often serving as second-line treatments, steroid-sparing agents, or in combination with biologics to enhance efficacy. However, their use is challenged by a narrow therapeutic window and significant interindividual metabolic variability, which increases the risk of drug-induced liver injury (DILI) [1,2,3]. Liver injury linked to thiopurines most often results from excessive methylation of 6-methylmercaptopurine nucleotides (6-MMPN), a dose-dependent, enzyme-driven process associated with biochemical cholestasis and elevated transaminases. Pharmacogenetic (PGx) testing for thiopurine S-methyltransferase (TPMT) and nudix hydrolase 15 (NUDT15) variants effectively prevents serious myelosuppression, but these static markers often do not predict hepatotoxicity, highlighting a significant genotype–phenotype gap [4,5]. This gap shows that liver toxicity is not determined by genetics alone but is strongly influenced by dynamic, context-dependent factors. These include secondary mtabolic pathways, drug–drug interactions (DDI), inflammation, and the regulatory roles of pro-inflammatory cytokines and cytochrome P450 (CYP) enzymes. These factors can cause rapid, clinically significant shifts in metabolic phenotype (phenoconversion), complicating the prediction of toxicity and the optimization of therapy [6]. Therefore, understanding these multilayered mechanisms—including transporter function, gene–environment interactions, and the hepatic metabolic network—is essential for advancing individualized risk assessment and precision medicine in therapy [7].

6-MP is a cytostatic prodrug that is converted into active thioguanine nucleotides (6-TGNs) through a series of metabolic steps. These nucleotides are incorporated into DNA and RNA, leading to the inhibition of nucleic acid synthesis and cell proliferation, particularly in rapidly dividing hematopoietic cells. TPMT catalyzes the S-methylation of 6-MP to 6-methylmercaptopurine (6-MMP). 6-MMP is hepatotoxic at high concentrations. Loss-of-function variants in *TPMT *(e.g., *2, *3A, *3B, *3C) reduce or abolish enzyme activity, leading to excessive 6-TGN accumulation and an increased risk of myelosuppression [5,8,9]. NUDT15 is a crucial enzyme that hydrolyzes cytotoxic 6-thioguanine triphosphate (6-TGTP) to the monophosphate form, preventing excessive incorporation of 6-TGNs into nucleic acids. Loss-of-function NUDT15 variants (notably the R139C allele, which is especially prevalent in East Asian populations) lead to increased sensitivity to thiopurines and an increased risk of severe, early leukopenia and alopecia [4,10,11].

MTX is a folate antagonist that inhibits dihydrofolate reductase (DHFR), thereby blocking tetrahydrofolate synthesis and, consequently, DNA synthesis and repair [12,13]. MTX is primarily metabolized by aldehyde oxidase (AOX), which converts MTX to 7-hydroxymethotrexate, a less active but potentially nephrotoxic metabolite. MTX is converted into polyglutamated forms (MTX-PGs) that are retained within cells, enhancing the inhibition of folate-dependent enzymes [14].

Both thiopurine and MTX, as well as their metabolites, are substrates for hepatic and renal transporters. ABCC2 (MRP2) and ABCC4 (MRP4) mediate the efflux of MTX and its polyglutamates, as well as 6-MP metabolites, from hepatocytes and renal tubular cells. SLCO1B1 (OATP1B1) mediates hepatic uptake of MTX [12,13,14,15,16,17]. Reduced-function *SLCO1B1* alleles (e.g., *5, *15) are associated with delayed MTX clearance and increased risk of toxicity [15,17]. These transporter-related variants, along with enzymatic and environmental factors, complicate dose optimization and patient outcome prediction during combination therapy.

In thiopurine metabolism, TPMT competitively produces the hepatotoxic metabolite 6-MMP; at the same time, inflammation-induced downregulation of CYP gene expression reduces metabolite clearance [6,18]. Similarly, intracellular polyglutamated MTX forms inhibit folate metabolism targets, and decreased CYP enzyme expression can hinder rapid elimination, worsening toxicity.

Pro-inflammatory cytokines such as IL-6 and TNF-α play a key role in modulating hepatic drug metabolism and disposition [19]. These cytokines directly repress CYP gene expression by interfering with ligand-activated nuclear receptors (such as pregnane X receptor (PXR) and constitutive androstane receptor (CAR)) and hepatic transcription factors, including HNF4α [20,21]. This inflammation-induced downregulation not only reduces the activity of drug-metabolizing enzymes but also suppresses the expression of key hepatic and renal transporters. As a result, drug clearance is diminished, leading to increased systemic and intracellular exposure to agents such as 6-MP and MTX. This phenomenon, known as phenoconversion, results in a mismatch between the genotype-predicted and the actual metabolic phenotype. The combined effects of genetic polymorphisms and cytokine-driven suppression of enzymes and transporters significantly elevate the risk of hepatotoxicity and myelosuppression, and increase the likelihood of clinically relevant drug–drug interactions, particularly during combination therapy [3,12,19,22]. PGx testing is now recommended by major guidelines (CPIC, DPWG, etc.) for both thiopurines and MTX [8,23]. Panel-based or sequencing assays commonly test for actionable variants in: *TPMT *(*2, *3A, *3B, *3C); *NUDT15* (*notably R139C*); *SLCO1B1* (*5, *15) and *CYP2C9* (*2, *3) [4,9,10,15,24].

These tests enable pre-therapeutic risk stratification and dose adjustment to mitigate severe toxicity. However, while *TPMT* and *NUDT15* genotyping reliably predict thiopurine-induced myelosuppression, these markers have limited value in predicting hepatotoxicity, underscoring the genotype–phenotype gap.

In modern ALL and IBD management, polypharmacy is common, and patients are often exposed to additional hepatotoxic or hepatically metabolized agents such as antibiotics, antifungals, and biologic therapies [25]. These exposures add complexity because they may alter hepatic enzyme activity or compete for metabolic pathways, increasing the risk of DLI. Both genetic and non-genetic factors (inflammation, DDIs, epigenetic modulation) interact to shape drug response, often leading to phenoconversion and unpredictable toxicity profiles. This complexity highlights the limitations of relying solely on static genomics and underscores the need for dynamic, multi-omics approaches and vigilant clinical/laboratory monitoring to optimize therapy and prevent adverse outcomes [26,27]. Polypharmacy (common in ALL and IBD) can alter CYP enzyme activity (CYP1A, CYP2D6, CYP2C9, CYP3A4, CYP2E1), with direct effects on MTX metabolism and indirect effects on thiopurine [19,28,29,30,31]. CYP enzymes are responsible for metabolizing many co-administered medications, and variations in CYP activity can significantly impact overall hepatic metabolic capacity. In cases of polypharmacy, competition for CYP-mediated metabolism may lead to metabolic bottlenecks, the buildup of toxic intermediates, and an increased risk of liver injury [25].

### Aims

This review advocates an integrated metabolic-inflammatory perspective that, beyond polygenic panels, employs a multidimensional diagnostic and therapeutic approach to enhance the efficacy of **thiopurine–methotrexate combination therapy** while significantly reducing toxicity. We provide a detailed overview of the hepatotoxic effects of thiopurine and MTX combination therapy, **focusing on the pharmacogenetic and metabolic complexities** underlying these adverse outcomes. We examine in detail the contribution of primary metabolic enzymes—TPMT and NUDT15—and their well-characterized polymorphisms, as well as secondary CYP-mediated pathways (CYP2C9, CYP2D6, and CYP3A4) and the impact of drug interactions on these systems. The review highlights how co-administered drugs, the immunobiology of inflammation, and environmental factors interact with or influence **the genetic background at the metabolomic level**, thereby shaping the dynamic liver environment and **modulating the risk of DILI**.

A key novelty of the review is its forward-looking focus on the development and implementation of multidimensional models for risk prediction and therapeutic monitoring. We hypothesize and recommend that future research place greater emphasis on integrating PGx data, inflammatory biomarkers, DDI profiles, and metabolite levels, leveraging multi-omics approaches, to enable more accurate, personalized risk assessment and real-time therapeutic guidance. Such systems could significantly improve the efficacy and safety of personalized therapy by accounting for both dynamic biochemical states and genetic background, thereby bridging the gap between PGx prediction and clinical outcomes.

In addition, we address several currently limiting gaps in the field of hepatotoxicity, including the clinical relevance of metabolic shunts, the genetic and metabolic effects of secondary genes, the **genetic and metabolic basis of phenoconversion**, and the precise role **of inflammation-mediated modification** of enzyme activity. The development of multi-omics platforms to comprehensively characterize these complex interactions will be crucial for reducing the risk of hepatotoxicity and addressing synergistic drug–drug and drug-inflammation interactions.

Ultimately, by elucidating these multi-level, complex mechanisms and advocating strategies, this review aims to inspire new approaches and research directions that will define the next era of precision medicine in ALL and IBD therapy, and beyond.

## 2. Materials and Methods

A comprehensive literature search was conducted to identify relevant studies exploring the synergistic hepatotoxicity of thiopurine and MTX co-therapy in several diseases. Systematic searches were performed across multiple electronic databases, including PubMed/MEDLINE, Scopus, Web of Science, and Google Scholar, covering the period from January 2000 to March 2026.

The search strategy employed a combination of Medical Subject Headings (MeSH) terms and specific keywords. The primary search strings included: “6-mercaptopurine” OR “6-MP”; “thiopurines”; “methotrexate”; “drug induced hepatotoxicity”, OR “DILI”); “pharmacogenetics”; “PGx”; (“TPMT” OR “NUDT15” OR “SLCO1B1” OR “ABCC2” OR “ABCC4”); “inflammation”; “cytokines” (“IL-6” OR “TNF-alpha”); “acute lymphoblastic leukemia” OR “ALL”; “IBD” OR “inflammatory bowel diseases”.

Cumulative risk estimates were derived using a multiplicative model of independently replicated Odds Ratios (ORs) from the literature, assuming no linkage disequilibrium between the selected loci. Both absolute and relative risk estimates were derived to quantify inter-genotypic differences in hepatotoxicity. Traditional statistical computations and forest plots were generated using GraphPad Prism 10.

In accordance with the journal’s policy, Generative AI was utilized solely for technical assistance in text structuring, linguistic refinement, and scientific formatting to enhance the clarity and academic quality of the manuscript. The conceptual framework, data interpretation, and final scientific conclusions remain the sole responsibility of the authors.

## 3. Results

Our findings underscore that, beyond the established utility of *TPMT* and *NUDT15* genotyping to prevent myelosuppression, secondary metabolism mediated by CYP enzymes is a critical, under-recognized factor in the safety and efficacy of therapy. CYP isoforms—particularly CYP2C9, CYP2D6, and CYP3A4—not only contribute to the oxidative biotransformation of thiopurine, such as 6-MP and MTX, but are also subject to dynamic regulation at the transcriptional level. Pro-inflammatory cytokines such as IL-6 and TNF-α, as well as exogenous compounds, can modulate CYP gene expression via ligand-activated nuclear receptors, including the PXR and CAR, and hepatic transcription factors such as HNF4α [18,19,21,28,29]. These signaling pathways mediate crosstalk between inflammation, xenobiotic sensing, and metabolic adaptation, leading to transcriptional repression or activation of CYP genes [19].

### 3.1. The Role of CYP Polymorphisms as Secondary Risk Factors

International guidance, such as CPIC and DPWG, has developed robust pharmacogenomic guidelines for thiopurine dosing based on the functional impact and population frequency of variant alleles in *TPMT* and *NUDT15* [8,10,23,24]. Polymorphisms are classified into (1) common, well-characterized alleles (e.g., TPMT*2, *3A, *3B, *3C, and NUDT15*3) that strongly reduce enzyme activity and account for most variant alleles detected in clinical practice, and (2) include rarer or less well-defined variants. According to these guidelines, patients are categorized as normal, intermediate, or poor metabolizers based on genotype, with dosing recommendations ranging from standard initiation to substantial dose reduction or alternative therapies for poor metabolizers. Notably, individuals with intermediate TPMT and NUDT15 function require further dose reductions and close monitoring. While these recommendations have improved the safety and efficacy of thiopurine therapy, emerging data underscore that secondary risk factors such as CYP polymorphisms must also be considered to fully capture individual susceptibility to adverse effects during combination therapy. CYP polymorphisms, drug interactions, and inflammatory processes indirectly influence the efficacy of 6-MP and MTX maintenance therapy and contribute to liver toxicity by altering the hepatic metabolic environment. In patients with reduced-function CYP alleles (e.g., CYP2C9*2, *3), drugs that inhibit or induce CYP activity decrease hepatic metabolic capacity. Inflammation-mediated downregulation of CYP gene expression further reduces hepatic metabolic capacity. These factors may impair the clearance of MTX and its polyglutamated metabolites and modify the secondary metabolism of thiopurines, despite them not being a direct CYP substrate. Consequently, systemic and intrahepatic exposure to both drugs and their potentially toxic metabolites increases. Inflammation and CYP inhibition may also redirect metabolism toward alternative pathways, such as TPMT-mediated methylation or transporter-mediated efflux, leading to accumulation of hepatotoxic compounds, such as 6-methylmercaptopurine (6-MMP), or delayed MTX elimination. This complex metabolic interplay raises the risk of dose-dependent liver toxicity, unpredictable drug responses, and additive or synergistic toxicity during combination therapy. These findings highlight the need for an integrated multi-omics risk assessment that incorporates genetic, pharmacological, and physiological factors to optimize the safety and efficacy of thiopurine and MTX treatments.

In this context, fluctuations in disease activity can dynamically change hepatic drug metabolism, leading to periods of higher vulnerability to toxicity. For instance, during IBD flares or systemic infections, reduced CYP activity may impair the metabolism of other medications, thereby increasing the liver’s overall metabolic load and, in turn, worsening thiopurine-related hepatotoxicity. Furthermore, this effect is influenced by circadian timing, with more pronounced CYP suppression at certain times of day, suggesting that both disease activity and the timing of doses may affect drug effectiveness and toxicity. These findings emphasize the importance of considering not only static pharmacogenetic factors but also fluctuating inflammatory states when evaluating drug metabolism and toxicity risk in IBD patients.

#### 3.1.1. Modification of CYP Gene Expression

CYP gene expression is tightly regulated by a network of ligand-activated nuclear receptors, most notably the PXR (NR1I2) and the CAR (NR1I3), as well as hepatic transcription factors such as hepatocyte HNF4α [30,31]. Under physiological conditions, these nuclear receptors function as cellular sensors of endogenous metabolites and xenobiotics, including drugs, environmental chemicals, and bile acids. Upon ligand binding, PXR and CAR translocate to the nucleus, where they heterodimerize with the retinoid X receptor (RXR) and bind to specific response elements in the promoter regions of CYP genes—primarily those encoding CYP3A4, CYP2C9, and CYP2B6—thereby inducing their transcription [32,33,34,35]. HNF4α, a main regulator of hepatic gene expression, interacts with nuclear receptors and other co-regulators to establish the basal and inducible expression of a broad range of genes involved in drug metabolism and transport, including several CYP isoforms. Crosstalk between these transcriptional regulators is further modulated by inflammatory signaling: pro-inflammatory cytokines (e.g., IL-6, TNF-α, IL-1β) activate intracellular pathways (such as JAK/STAT and NF-κB) that can directly or indirectly interfere with nuclear receptor activity or reduce HNF4α expression [20,30,36].

Chromatin immunoprecipitation (ChIP) and complementary DNA–protein assays show that activation of inflammatory signaling (e.g., TNF-α) increases recruitment of NF-κB p65 to CYP3A4 regulatory regions and, at the same time, disrupts PXR·RXRα association with those sequences—a mechanism that directly suppresses CYP3A4 transcription [37].

In primary human hepatocytes IL-6 exposure decreases PXR levels and PXR-dependent transactivation, and PXR has been shown to be necessary for the full IL-6-mediated down-regulation of CYP3A4, consistent with loss of PXR occupancy/function after cytokine challenge.

The interplay among PXR, CAR and HNF4α with cytokine signaling can be established with rigorous, locus-specific and time-resolved measurements that link molecular mechanism to functional outcome.

Functional readouts include PXR/CAR receptor-mediated reporter assays (PBREM/XREM), electrophoretic mobility shift assay (EMSA), and co-immunoprecipitation to assess receptor·RXR and DNA binding disruption. They also include probe-substrate enzyme activity measurements, such as midazolam for CYP3A4 and bupropion for CYP2B6, and in vitro and in vivo pharmacokinetic data (clearance, AUC). These are essential for translating molecular interactions into phenoconversion. Causality and reversibility may be shown by inhibiting the STAT3/NF-κB pathway, using genetic manipulation, overexpressing PXR/CAR/HNF4α, or introducing stabilizing mutants. These interventions are expected to restore receptor occupancy, reduce post-translational modifications, reestablish target gene expression, and normalize enzyme activity and pharmacokinetic parameters. Together, these coordinated molecular and functional analyses provide robust and reproducible evidence that cytokines directly modulate nuclear receptor signaling, altering hepatic detoxification dynamics. This is particularly relevant for drug combinations such as 6-MP and MTX.

This multilayered regulation is central to understanding how **gene–environment interactions** and disease states can **induce phenoconversion, leading to clinically significant changes in drug clearance, efficacy, and toxicity profiles**, particularly in the context of complex therapies such as combined 6-MP and MTX treatment.

#### 3.1.2. The Effect of CYP Variants on Toxicity by Thiopurine and MTY Therapy

Reduced-function *CYP2C9* variants, such as the *2 (rs1799853) and *3 (rs1057910) alleles, significantly impair hepatic drug metabolism by reducing the oxidative capacity of the enzyme [32,38]. Coadministration of MTX in individuals with these genetic variants leads to accumulation of MTX and its metabolites, increasing exposure to potentially hepatotoxic intermediates and the risk of liver injury [12,33,39]. This molecular effect is further exacerbated by gene-environment interactions, including DDIs and inflammatory conditions, which downregulate CYP2C9 expression through cytokine-mediated suppression of nuclear receptors such as PXR and CAR, as well as hepatic transcription factors. For example, during systemic inflammation, the IL-6/STAT3 pathway is activated: IL-6 binds to its hepatocyte receptor, triggering JAK-mediated phosphorylation and dimerization of STAT3, which translocates to the nucleus and directly or indirectly represses HNF4α expression [36,40,41]. *CYP2C9* genotyping may be considered in specific populations, as reduced-function alleles can impair MTX elimination. However, routine PGx-guided dosing of MTX based solely on *CYP2C9* or other *CYP* variants is not universally recommended, given the predominant role of non-CYP pathways (e.g., aldehyde oxidase) in MTX metabolism [42,43].

CYP3A4*22 (rs35599367) variant affects the metabolism of many xenobiotics, including corticosteroids, which are commonly used in treatment protocols [24,44]. Changes in the expression or activity of these enzymes can destabilize systemic corticosteroid levels, modify immune responses, and, indirectly, increase the risk of adverse reactions (e.g., DILI) [45]. Furthermore, pro-inflammatory cytokines such as TNF-α and IL-1β activate the NF-κB pathway, and the p65 subunit can interact with PXR in the nucleus, preventing it from heterodimerizing with RXR and binding to the *CYP3A4* promoter region. This diverts PXR from its metabolic gene targets, which further reduces CYP expression during inflammation.

Among the *CYP2E1* polymorphisms, CYP2E1*5B (rs2031920) and CYP2E1*6 (rs6413432) are associated with increased inducibility and increased enzyme activity [46,47]. In systemic inflammation, elevated TNF-α levels increase CYP2E1 expression, thereby stimulating oxidative metabolism. This process reduces glutathione (GSH) levels in cells via reactive oxygen intermediates, thereby weakening cellular antioxidant defenses. The resulting oxidative stress enhances MTX cytotoxicity and exacerbates liver cell damage [48].

CYP2E1 generates reactive oxygen species under inducible conditions and may contribute to GSH depletion and increased MTX cytotoxicity. However, evidence directly linking common *CYP2E1* polymorphisms or systemic TNF-α elevations to clinically significant oxidative stress and liver injury remains inconclusive. Definitive causal inference requires concurrent measurement of CYP2E1 activity, reactive oxygen species (ROS), and GSH dynamics, along with functional inhibition or genetic validation.

Together, these CYP-mediated secondary mechanisms demonstrate how genetic variation, inflammatory regulation of gene expression, and metabolic interactions converge to influence the toxicity in combination therapy.

### 3.2. Inflammation-Induced Phenoconversion

The clinical efficacy and safety of thiopurine and MTX co-therapy are influenced not by isolated genetic or pharmacokinetic factors but by a dynamically regulated, complex metabolic network. Proinflammatory cytokines IL-6, TNF, and IL-1β serve as key regulators of hepatic drug-metabolizing capacity. They suppress the expression and activity of CYPs (CYP3A4, CYP1A2, and CYP2C9) and essential hepatic transporters (e.g., SLCOs, ABCC4) through transcriptional and post-transcriptional mechanisms [15,17,49]. This molecular interference leads to **inflammation-mediated phenoconversion**, a transient state where patients genotypically classified as normal metabolizers (gNM) clinically behave as poor metabolizers (fPM). Acute or chronic inflammatory episodes, such as IBD flares or systemic infections, significantly alter intracellular exposure and metabolite ratios of thiopurine and MTX. These changes often correlate with elevated C-reactive protein (CRP) and cytokine levels [18,50]. Consequently, these biochemical parameters serve as reliable biomarkers for the progressive decline in drug-metabolizing capacity and the risk of increased intrahepatic drug concentrations. Phenoconversion results in an increased risk of DILI and reduced therapeutic efficacy. Reduced expression of efflux transporters (e.g., MRP2/ABCC2 and BSEP) leads to accumulation of toxic intracellular conjugates, including 6-MMP and endogenous bile acids, resulting in cholestasis and hepatocellular stress. Inflammatory cytokines also worsen liver injury by inducing cellular apoptosis and TGFB1-mediated activation, accelerating liver fibrosis and chronic vascular lesion development. In the context of 6-MP/MTX co-therapy, these interactions may impair detoxification pathways, such as by depleting glutathione, and disrupt the 6-TGN/6-MMP balance, potentially increasing the risk of severe adverse effects in certain phenotypes [51,52]. These findings support moving from static, genotype-only protocols to adaptive, multiparametric methods that integrate PGx with real-time inflammatory biomarkers (CRP, IL-6) and dynamic therapeutic drug monitoring (TDM) [53]. Effective management of inflammatory processes is essential to reverse phenoconversion and maintain precision dosing amid the patient’s changing metabolic state.

### 3.3. The Role of Transporters

In the combination therapy of 6-MP and MTX, transmembrane transporters are essential yet often overlooked regulators of drug distribution and hepatotoxicity risk. These transporters, including solute carrier (SLC) uptake systems such as SLC19A1 (rs1051266) and SLCO1B1 (rs4149056), and ATP-binding cassette (ABC) efflux pumps like ABCC2 (MRP2; rs717620 (-24C>T) or rs3740066 (3972C>T) or 1774del) and ABCC4 (MRP4; rs2274407 or rs11568658 or rs3765534), influence the intracellular environment by regulating drug entry into hepatocytes and the retention and elimination of cytotoxic metabolites [33,54,55]. Recent studies show that genetic polymorphisms in these transporter genes, especially SLCO1B1 c.521T>C and functional impairments in ABCC2/ABCC4 (rs717620/rs2274407), may disconnect (local uncoupling) hepatic drug accumulation from systemic drug levels, explaining the “silent” yet severe localized DILI despite normal serum monitoring (Table 1) [55].

It has been demonstrated that the regulatory role of these transporters extends beyond static genetically predicted risk factors. In particular, during systemic inflammation, IL-6, TNF-α, and IL-1β inhibit the transcription and membrane localization of key hepatic transporters [6,19,56,57]. This creates a “closed transporter” hepatocyte state, leading to phenoconversion and increasing the risk of toxic metabolite accumulation, especially in patients with a high TPMT phenotype, metabolic shunts, and drug–drug interaction regimens. Thus, liver injury during 6-MP/MTX combination therapy should be considered a dynamically formed and regulated, multilayered metabolic network, in which, in addition to primary and secondary genes, **genetic variants of transporters, the pharmacological environment, and the organism’s current inflammatory state collectively influence risk and outcomes**.

This complex multi-omics approach highlights the need for an integrated, systems-level diagnostic and therapeutic approach that goes beyond and complements traditional pharmacogenetics [26,42]. **Safe and effective 6-MP/MTX therapy requires a **combination of** expanded pharmacogenomic profiling, real-time monitoring of inflammatory biomarkers, and therapeutic drug and metabolite surveillance**. Addressing the multidimensional, dynamic regulation of hepatic transporters is essential for the full implementation of precision medicine and for reducing liver injury in pediatric oncology and inflammatory bowel disease care.

**Table 1:** Primary and Secondary pharmacogenetic variants for optimizing 6-MP and MTX co-therapy and minimizing hepatotoxicity in ALL Maintenance. Hepatotoxicity-focused OR columns estimate relative risk specifically for liver adverse events (ALT/AST elevation, cholestasis, clinically significant hepatic injury, progression to fibrosis) rather than combined marrow + liver endpoints. OR ranges are approximate, mechanistically informed, and multiplicative; actual patient risk depends on zygosity (hetero vs. homo), drug dose, renal function, co-medications, inflammatory status, and clinical context. Variants and functional effects shown are evidence-informed but not uniformly prospectively validated for a composite hepatotoxicity score.

Priority Level	Gene(s)	Key Variants	Clinical Significance and Biological Rationale	Refs
*Primary*				
	*TPMT*	rs1142345, rs1800460, rs1800462	Deficiencies lead to the toxic accumulation of 6-TGN, causing severe, potentially myelosuppression.	[5,8,58]
	*NUDT15*	rs116855232 (R139C)	Variants are strongly associated with severe thiopurine-induced leukopenia and alopecia, particularly in Asian and Latin American populations.	[4,8]
*Secondary*				
Thiopurine Pathway	*ITPA*	rs1127354 (94C>A)	*ITPA* deficiency leads to the accumulation of thio-ITP, which is associated with idiosyncratic adverse reactions and increased metabolite-related toxicity.	[59,60]
Folate/MTX Pathway	*MTHFR*	rs1801133 (677C>T), rs1801131 (1298A>C)	These polymorphisms modulate the efficiency of the folate cycle and the one-carbon metabolism pathway. They serve as critical pharmacodynamic markers for methotrexate sensitivity and toxicity.	[61,62]
Liver Health	*PNPLA3*, *TM6SF2*	rs738409, rs58542926	These variants are independent predictors of baseline susceptibility to hepatic steatosis and fibrosis. Pre-existing liver vulnerability can exacerbate DILI during intensive maintenance therapy.	[63]
Uptake Transporters	*SLC19A1*, *SLCO1B1*	rs1051266, rs4149056 (c.521T>C)	Variants in *SLCO1B1* are known to alter drug uptake, leading to localized metabolic imbalances and increased toxicity risk.	[15,17,49]
Efflux Transporters	*ABCC2*, *ABCC4*, *ABCB1*	rs717620, rs1751034, rs1045642	Functional deficits trigger intracellular sequestration of 6-MMP and other hepatotoxins, promoting cholestatic injury.	[17,55]
Metabolic Modulators	*CYP2C9*, *CYP2D6*	rs1799853 (*2), rs1057910 (*3), rs3892097 (*4)	Variants increase the risk of metabolic bottlenecks during polypharmacy, leading to phenoconversion and synergistic hepatotoxicity.	[32,47,57]
Oxidative intermediers	*CYP2E1*	rs2031920 (*5B), rs6413432 (*6)	Elevated TNF-α induces CYP2E1 activity, increases ROS production. Variants with higher inducibility exacerbate MTX cytotoxicity (up to 2.9-fold) by depleting the cellular GSH pool.	[47,64]

### 3.4. Rationale for an Expanded Genetic Panel: Synergistic Risk Assessment and Molecular Synthesis

#### 3.4.1. Synergistic Gene Combination

The extent of hepatotoxicity during 6-MP and MTX combination therapy is determined not only by isolated genetic variants but also by their synergistic, multiplicative interactions. Our results highlight that homozygous variants of the primary genes recorded in current guidelines, such as *NUDT15* (rs116855232) and *TPMT* (rs1142345, rs1800460, rs1800462), are critical risk factors for hepatotoxicity. This association stems from insufficiency of primary detoxification pathways (methylation and dephosphorylation), leading to increased accumulation of cytotoxic 6-TGN and disruption of metabolic balance. The analysis also highlights that secondary variants that regulate hepatic exposure, such as *SLCO1B1* (rs4149056) and *ABCC2* (rs717620), increase risk and modify the cellular uptake and biliary excretion of MTX and its conjugates, leading to intracellular toxic sequestration (Table 2). The key finding of the summary analysis and estimated toxicities is that extending the PGx panel to include secondary genes is justified by the significant increase in risk during combination therapy.

As shown in Table 2, if the primary *TPMT* or *NUDT15* homozygous defect is combined with secondary transporter variants (e.g., SLCO1B1, ABCC2) and the homozygous polymorphism of *PNPLA3* (rs738409), which affects the liver’s regenerative capacity, the risk of hepatotoxicity rises to a high level (Table 2). In this context, steatosis and fibrosis induced by PNPLA3 synergize with transporter retention, increasing the risk of chronic progression by three to six times beyond acute liver parenchymal damage. Similarly, the presence of homozygous NUDT15 and MTHFR variants (rs1801133) shows stronger synergy in which nucleotide metabolism disorders and folate pathway damage together increase metabolic and mitochondrial stress in liver cells (Table 1 and Table 2). The importance of polypharmacy and secondary oxidative pathways is shown by the combination of homozygous TPMT variants and reduced-function alleles of CYP2C9*2 and *3 (rs1799853, rs1057910), as well as variants of *CYP3A4/3A5* (rs35599367) and *CYP2D6 *(rs3892097) (Table 2). In these cases, pharmacological phenoconversion due to reduced CYP enzyme capacity and insufficient clearance of co-medications indirectly worsens thiopurine-induced DILI. These data clearly show that testing focused solely on a single gene fails to capture the “synergistic multiplier” effect. Therefore, using an expanded genetic panel may be justified to preemptively identify high-risk patient groups and ensure the safety of precision medicine care.

#### 3.4.2. DDI Enhancing Hepatotoxicity

Polypharmacy is a clinical necessity to prevent complications, which fundamentally determines the safety of 6-MP and MTX therapy. Polypharmacy is common, and patients are frequently exposed to additional hepatotoxic or hepatically metabolized agents, including antibiotics, antifungals, and biologic therapies. These exposures further increase complexity by altering hepatic enzyme activity or competing for metabolic pathways, thereby increasing susceptibility to DILI. As such, thiopurine-related hepatotoxicity should increasingly be viewed not as an isolated drug effect, but as the result of a dynamic integration between drug metabolism, co-medications, and host factors, underscoring the need for integrated therapeutic drug monitoring and individualized risk assessment.

Complications of acute infections in immunosuppressed patients, such as protection against bacterial infections or mandatory prophylaxis against Pneumocystis jirovecii pneumonia, require the use of drugs such as trimethoprim-sulfamethoxazole (TMP-SMX). Similarly, invasive therapy of acute fungal infections occurring during treatment often requires the administration of azole-type antifungal agents, such as fluconazole or ketoconazole [65,66]. Although these drugs are essential for patient survival, they impose an increased burden and congestion on the metabolic networks of the liver and kidneys, as they compete for pathways responsible for the elimination of thiopurin and MTX. The presence of polypharmacy—observed in the vast majority of DILI cases—thus creates a dynamic interaction network in which the necessary co-medications indirectly amplify the hepatotoxic potential of chemotherapeutic agents [39,67,68]. Among the interactions relevant in clinical practice, the effects of sulfonamides and antifungal agents stand out, which synergize with inflammation-induced phenoconversion and carry an extreme risk of toxicity. The use of TMP-SMX directly inhibits the renal clearance of MTX by competing with renal transporters (e.g., MRP2), which leads to systemic MTX accumulation and increased liver cell damage [69]. In parallel, fluconazole, as a strong CYP2C9 inhibitor, prevents the efficient breakdown of co-medications and “shunts” the metabolism of 6-MP towards the toxic 6-MMP, even in patients with normal genotypes. Calculated data demonstrate that synergy (6-MP + MTX + sulfonamide/antifungal agent) can increase the risk of serious adverse events by up to 95-fold in patients with NUDT15 or TPMT variants. Therefore, in the treatment of acute infections, it is essential to supplement static PGx data with real-time monitoring: tracking inflammatory markers (CRP, IL-6) and metabolite levels (6-TGN, 6-MMP) allows for precise dose recalibration in the unstable metabolic environment induced by polypharmacy [39,45,69].

### 3.5. Synergy of Secondary Genetic Variants and Polypharmacy

The clinical signs of hepatotoxicity during the treatment of ALL and chronic autoimmune diseases like IBD and rheumatoid arthritis (RA) stem from a complex interplay of genetic susceptibility, inflammatory signaling, and drug interactions, rather than from static genetic inheritance alone. Inflammation-driven phenoconversion plays a key role in this process. Systemic cytokines, such as IL-6 and TNF-α, which are consistently elevated in active IBD and ALL, inhibit transcription of essential hepatic transporters, including SLCO1B1 and ABCC2. This can cause a genotypically “wild-type” patient to become functionally impaired. The use of prophylactic agents, especially sulfamethoxazole (SMZ) or sulfonamides (TMP-SMX) to prevent opportunistic infections during heavy immunosuppression, further worsens this condition. SMZ competes with renal and hepatic organic anion transporters (OATs), potentially decreasing MTX clearance by about 38.7%, similar to other competitive inhibitors such as penicillin. These external factors work together with high clinical hepatic risk score (CHRS) variants, including *SLCO1B1* mutations and methylenetetrahydrofolate reductase (MTHFR) polymorphisms, to promote toxic molecular shunting toward secondary, more damaging metabolic pathways. In thiopurine therapy, this destabilization is especially dangerous because MTX-related metabolic stress can upset the balance between 6-TGN and 6-MMP metabolites, leading to intrahepatic accumulation and localized tissue damage. Furthermore, age-related and weight-related physiological maturity must be incorporated into risk models, since MTX clearance is markedly lower in adults (0.11 L/kg/h) than in children (0.23 L/kg/h).

Enhancing risk model accuracy requires precise integration of physiological covariates, including age, body surface area (BSA), and renal function. MTX clearance varies with age: in children, the average clearance is 0.23 L/kg/h, while in adults it decreases to 0.11 L/kg/h. This age-related difference, due to physiological maturation, leads to a lower toxicity threshold in adult IBD patients [70]. Consequently, adults show increased susceptibility to DILI, even when genetic and inflammatory profiles are similar.

A predictive clinical approach requires dynamic modeling that considers metabolic disturbances from inflammation, along with real-time changes in renal function (such as glomerular filtration rate, GFR) and BSA as interconnected, personalized safety factors rather than isolated parameters.

**Figure 1:**
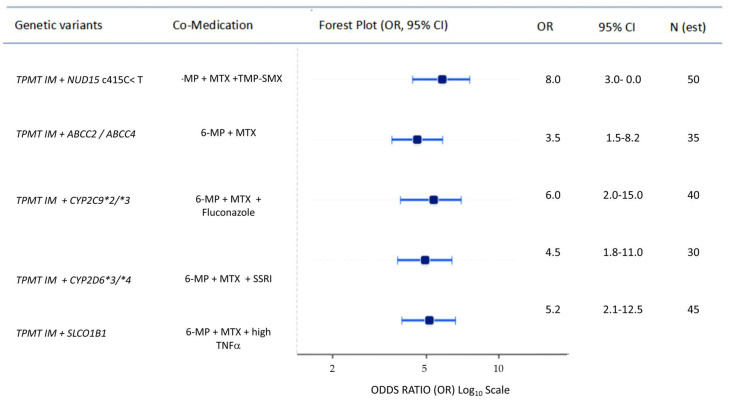
Estimated hepatotoxicity risk (odds ratios) for 6-MP + MTX co-therapy across combined pharmacogenetic and co-medication constellations.

The most severe risk of liver damage occurs with combined excretory blockade and primary metabolic pathway failure. When a patient carries *TPMT* and/or NUDT15 variants (e.g., rs1800460, rs116855232) alongside the *SLCO1B1* (rs4149056) polymorphism, the balance of cellular adsorption and efflux is disrupted. In this genetic context, administering sulfonamides (e.g., TMP-SMX) during therapy may inhibit renal excretion of MTX and block transporters, thereby significantly increasing the risk of hepatotoxicity (Figure 1) [3,69,71]. The diagnostic challenge is compounded by hidden phenoconversion, where patients with genotypically normal TPMT activity may become critically ill. Additionally, patients with *CYP2C9* gene variants showing reduced phenotypic activity (rs1799853, rs1057910) who receive co-medications such as the antifungal fluconazole experience inhibition of secondary metabolic pathways (Figure 1) This shifts 6-MP metabolism toward the hepatotoxic 6-MMP, leading to extreme 6-MMP accumulation and direct parenchymal damage, even in individuals with low genetic risk [18,39]. This process may worsen with activation of the oxidative stress axis. In patients with the CYP2E1*5B (rs2031920) variant, MTX treatment becomes particularly toxic in an inflammatory environment with elevated TNFα levels (Figure 1). Cytokine-induced enzyme activity increases and depletes hepatic GSH stores, accelerating hepatocyte apoptosis and the development of chronic fibrosis.

These findings indicate that precision oncology protocols should extend beyond monitoring *TPMT * and *NUDT15* variants when considering hepatotoxicity as a treatment side effect.

Research results demonstrate that a multi-layered experimental strategy—combining time-resolved transcriptomic/proteomic profiling, functional enzyme assays, and pharmacokinetic readouts—can provide the specific, quantitative data needed to establish causal relationships and dynamics.

An integrated, multi-parametric approach that includes transporters, secondary CYP isoenzymes, and the dynamic inflammatory status significantly improves the predictability of hepatotoxicity. Achieving safe therapy is supported by the combined use of real-time biomarkers (such as CRP and cytokines) and an expanded pharmacogenomic panel, enabling individualized dose recalibration in the unstable metabolic environment induced by polypharmacy. To maximize the reliability of complex therapeutic contexts (e.g., 6-MP + MTX), we recommend performing preliminary clinical pharmacokinetic correlations in patients receiving 6-MP/MTX combined with laboratory CRP and IL-6 values.

The figure presents conceptual odds ratios (ORs, log10 scale) and approximate 95% confidence intervals for hepatotoxicity during 6-MP + MTX co-therapy under different gene–drug constellations.

The outcome: clinically significant toxicity (grade ≥ 3 neutropenia, ALT/AST ≥ 3× ULN, or DILI-related treatment discontinuation).

Reference: Wild-type genotype (TPMT*1/*1, NUDT15*1/*1, etc.) without interacting co-medication.

Statistics: point estimates and approximate 95% confidence intervals are expert-derived, literature-based assumptions (not obtained from a single cohort or meta-analysis), intended to visualize relative risk ranking rather than provide exact quantitative effect sizes. No formal regression modeling or meta-analytic pooling was performed.

All odds ratios are conceptual estimates derived from published pharmacogenetic and pharmacokinetic literature on thiopurines, methotrexate, and the respective co-medications. Values represent theoretical risk assessments based on a synthesis of independently reported genetic effect sizes. These findings are intended for risk stratification and hypothesis generation and require prospective clinical validation.

### 3.6. Dynamics of Clinical Biochemistry and Multigenic Synergy in Complex Polypharmacy

Hepatotoxicity related to the induction and intensive phases of ALL therapy, as well as autoimmune conditions like IBD, often appears as a hidden biochemical imbalance without symptoms. In these cases, asymptomatic increases in alanine aminotransferase (ALT) and aspartate aminotransferase (AST) often precede clinical signs of DILI, serving as early markers of underlying liver dysfunction that can significantly affect pharmacokinetics. Elevated ALT, in particular, is linked to impaired liver synthetic function—especially reduced albumin production—which then influences the volume of distribution and the binding of MTX in both blood and tissues. The complexity of polypharmacy, including drugs like thiopurines, MTX, and preventive SMZ, raises the risk of liver toxicity, especially in people with multiple high-risk genetic variants. For example, a patient with a harmful *SLCO1B1* gene variant—leading to a 35.3% decrease in MTX clearance—and simultaneous *ABCC2* deficiency will have reduced hepatic uptake and lowered efflux of MTX and its metabolites. This dual problem leads to a significant buildup of drugs within cells and increases metabolic stress, underscoring the importance of mechanism-based risk evaluation.

To shift from a reactive to a predictive approach to monitoring toxicity, clinical protocols should include highly sensitive, mechanism-based biomarkers alongside traditional liver function tests. The urinary coproporphyrin I/(I+III) ratio (DP3) is a strong, non-invasive marker for the activity of the MRP2/ABCC2 and, by extension, for the liver’s transporter-mediated clearance of MTX. Simultaneously tracking hematologic parameters—including hematocrit (HCT) and hemoglobin—is essential because MTX and its toxic metabolites tend to accumulate in red blood cells, and changes in HCT can significantly influence overall drug clearance and metabolite accumulation. Since renal elimination accounts for 70–90% of MTX removal, ongoing assessment of serum creatinine and creatinine clearance is crucial, especially when using polypharmacy with drugs like penicillins or sulfonamides that inhibit OAT. These interactions can reduce MTX clearance by up to 40%.

### 3.7. Expansion of Multimodal Pharmacogenetic and Systems-Level Models for Estimating Hepatotoxicity

Enhancing the safety of 6-MP and MTX co-therapy requires complex predictive models expansion that incorporates interactions among dynamic, mechanistic covariates, rather than relying solely on static descriptors. In this integrated framework, combined assessment of genetic polymorphisms—particularly TPMT, NUDT15, CYP enzymes, and SLCO and ABC transporters—provides a more accurate estimation of toxicity risk. The effects of “slow metabolizer” genotypes may be compounded by transporter limitations, such as reduced *SLCO1B1* or *ABCC2* activity, potentially decreasing hepatobiliary clearance of MTX and thiopurine metabolites.

Future models should incorporate cytokine-induced metabolic changes. Systemic inflammation and elevated IL-6 and TNF-α levels can modify drug processing capacity. A pro-inflammatory environment that downregulates transporter expression and alters plasma protein binding may cause genetically normal metabolizers to temporarily exhibit pharmacokinetic profiles similar to those of slow metabolizers. Including this dynamic in physiologically based pharmacokinetic (PBPK) models would enable the prediction of DDI effects from changes in cytokine levels [57,72].

A contemporary population pharmacokinetic (PopPK) approach should define clearance as a function that incorporates age-dependent physiological maturation and allometric scaling based on BSA [72]. It should pay particular attention to metabolic transitions between childhood and adulthood. The model structure should integrate nonlinear interactions among covariates, such as the synergy between BSA and renal clearance (GFR), using Bayesian statistical methods. This approach enables dynamic recalibration of DILI risk based on real-time clinical data, including serum creatinine and inflammatory markers.

To improve estimation accuracy, models should incorporate additional physiological factors such as sex and body weight. Faster baseline clearance linked to pediatric-specific characteristics is critical during ALL therapy. In adult IBD patients, physiological decline in renal function and steatosis may increase liver vulnerability. This refinement ensures the model describes not only population averages but also serves as a precision tool for predicting individual exposure changes during disease progression, aging, or cytokine elevation from infections. A systems-level approach can support clinical decision-making and optimal dosing.

### 3.8. Ecological Impact and Cost-Effectiveness of an Expanded Multimodal Integrated Pharmacogenomic Panel

This study evaluated the ecological and economic effects of integrating multi-gene PGx data, dynamic biomarkers, and model-based decision support for patients receiving 6-MP and MTX. The eco-score aggregates actionable genotypes, including *TPMT*, *NUDT15*, *ITPA*, *MTHFR*, *ABCC2*, *SLCO1B1*, and *CYP2C9*, to inform resource allocation [73]. Patients with high eco-scores received comprehensive PGx testing, intensive laboratory monitoring, and model-guided dose adjustments. Those with low scores received standard monitoring. The eco-score was calculated by assigning weighted points to high-risk alleles or haplotypes in each gene, reflecting both the number and clinical significance of actionable variants.

Pharmacogenomic-guided initial dosing, informed by expanded-panel results and electronic medical record (EMR)-embedded calculators, reduced the incidence of early severe hepatotoxicity in high-risk patients. Preemptive dose reductions of 25 to 75 percent, implemented when genotype and transporter risks coincided, led to fewer therapy interruptions and a lower incidence of grade 3 or higher transaminase elevations. Disease control was preserved in monitored groups. Real-time biomarker monitoring, including ALT/AST, bilirubin, GGT, 6-TGN, 6-MMP, MTX-PG, CRP, IL-6, and TNF-α, conducted at eco-score-adjusted intervals, provided early detection of hepatic stress and inflammatory phenoconversion [74]. This approach enabled timely, model-predicted dose modifications or brief therapy interruptions, thereby preventing progression to significant liver injury.

Extended PGx testing and eco-score–driven monitoring should first be implemented in patient groups with high risk and significant economic impact, such as pediatric ALL, adults with IBD on polypharmacy, and ethnicities with high allele prevalence. Access should expand incrementally as real-world outcome and cost–benefit data become available. Protocols should be continuously refined based on feedback from outcome metrics and economic analyses.

### 3.9. Conclusions for Clinical Integration

Current clinical management of 6-MP and MTX co-therapy primarily follows CPIC guidelines, which designate TPMT and NUDT15 genotyping as the gold standard. Testing these primary markers is essential to prevent dose-limiting myelotoxicity, but existing approaches have significant limitations in predicting hepatotoxicity. A critical literature review reveals no prospectively validated, consensus multigenic risk score that specifically predicts liver injury during combined 6-MP and MTX therapy. Much research relies on GWAS (Genome-Wide Association Studies) data, confirming the critical role of SLCO1B1 variants (e.g., rs4149056) in MTX clearance and delayed elimination, yet often analyzes pharmacokinetic endpoints in isolation. Furthermore, the validation of genetic risk factors for liver injury is frequently hindered by statistical constraints. As demonstrated in the MTX-DILI cohort study by Ektimmerman et al., the relatively small number of liver-specific cases—compared to more common toxicities such as myelosuppression—often limits statistical power to detect low-frequency variants with moderate effect sizes [75]. This inherent rarity and clinical heterogeneity of DILI underscore the limitations of relying solely on static, cohort-based GWAS and highlight the necessity of our proposed integrated systems pharmacology approach to capture subtle but clinically significant metabolic risks.

Future research should design a multicenter, prospective cohort study with sufficient power to evaluate genetic and clinical predictors of hepatotoxicity during combined 6-MP and MTX therapy. Integration of real-time pharmacogenomic testing and longitudinal monitoring would allow for validation of candidate risk variants and the development of a clinically actionable multigenic risk score.

Incorporating the functional roles of secondary genes identified by GWAS into diagnostic algorithms is crucial for scientific progress and to justify expanding the genetic panel. Transporter genetics (*ABCC2*, *ABCC4*, *SLCO1B1*) and hepatic parenchymal integrity variants (PNPLA3) are especially important because they influence the toxic burden on hepatocytes amid polypharmacy and synergistic DDI. Current models also fail to account for inflammation-mediated phenoconversion. GWAS data typically do not capture that a genotypically normal metabolizer may become a functional poor metabolizer due to cytokines (IL-6, TNF-α) released during the acute phase response. In clinical practice, this means a patient who genetically qualifies for a standard dose may experience unexpected toxicity or require dose adjustments during periods of inflammation, such as infection or chemotherapy-induced immunological stress. This dynamic condition limits static genetic tests, especially during infections in ALL maintenance therapy.

To enhance the clinical utility of hepatotoxicity prediction, future research must address the limitations of current GWAS, where the limited availability of liver-specific cases reduces statistical power. Prospective, multicenter validation studies should integrate an expanded pharmacogenomic panel—including GWAS-replicated markers of *MTHFR*, *ITPA*, and oxidative stress —with real-time clinical covariates. Advancement in this field requires developing a comprehensive Hepatotoxicity Risk Score that quantifies cumulative risk from molecular shunting and transporter blockade. This score should incorporate dose intensity, renal function, and dynamic inflammatory biomarkers such as CRP and IL-6. Achieving precision medicine in 6-MP/MTX therapy also requires a multi-omics approach that integrates transcriptomic and metabolomic data to account for both genotype and current immunometabolic status. Validation of these models across diverse ethnic populations and in real-world polypharmacy scenarios is essential to ensure risk stratification remains robust and reliable beyond traditional clinical trials.

### 3.10. Clinical Implementation: Advancing Innovation in 6-MP/MTX Therapy

Advancing clinical care requires integrating expanded PGx testing panels that assess multiple gene variants beyond *TPMT* and *NUDT15*, including *ITPA*, *MTHFR*, *ABCC2*, *SLCO1B1*, and *CYP2C9*. This approach enables individualized upfront dosing of 6-MP and MTX based on a composite genotype risk score, minimizing empirical titration. Combining PGx data with baseline liver function tests, dynamic biomarkers, and sequential metabolite monitoring helps clinicians identify patients at increased risk for myelotoxicity or hepatotoxicity early. It also helps optimize therapy through actionable, model-based dose adjustments. Incorporating transporter and CYP genotypes into PBPK and PopPK models enables simulation of drug–drug interactions, supports safer co-medication strategies, and enables proactive intervention. Clinical studies indicate that multi-gene PGx panels significantly improve toxicity prediction and reduce severe liver enzyme elevations. High-risk genotypes warrant initial dose reductions, which decrease therapy-related hospital admissions. Advanced, model-based recommendations updated in real time as new laboratory or biomarker data become available should integrate parameters such as GFR, BSA, ethnicity, and biomarker profiles to generate individualized dosing (Table 3). This is particularly important for high-risk subpopulations, including pediatric ALL patients, adults with IBD and polypharmacy, or ethnic groups with a higher prevalence of risk alleles. Embedding these data streams and models into electronic prescribing platforms will provide clinicians with genotype-guided dosing calculators, automated toxicity risk alerts, and longitudinal monitoring dashboards. Multidisciplinary collaboration is essential to standardize workflows, ensure real-time data integration and model validation, and support ongoing innovation through biobanking and shared data repositories.

To support clinicians in optimizing 6-MP/MTX therapy, consider measuring baseline CRP, IL-6, and metabolite levels (6-TGN, 6-MMP) before initiating or adjusting treatment. Where possible, repeat testing to confirm abnormal results and inform decision-making. If confirmed results show CRP above 30 mg/L and more than twice baseline, IL-6 above 30 pg/mL (or a locally relevant threshold), or a 6-MMP:6-TGN ratio above 20, consider temporarily reducing the dose or pausing therapy. For moderate findings (CRP or IL-6 10–30; 6-TGN 300–450; ratio 10–20), closer monitoring and a provisional dose reduction may be appropriate until confirmation (Table 3) [74,76,77]. If severe liver injury markers are present (ALT/AST above 3× ULN, bilirubin above 2× ULN, or any immediate action threshold), temporarily withhold treatment, confirm findings, consult hepatology, and resume with a conservative, genotype-guided dose reduction under close supervision. Local laboratories should establish assay-specific cutoffs and therapeutic ranges. This flexible, personalized approach can help clinicians optimize therapy, improve patient safety, and advance the management of 6-MP/MTX.

## 4. Discussion

Current clinical protocols for 6-MP and MTX maintenance therapy focus on preventing myelosuppression by screening TPMT and NUDT15 variants, as recommended by international guidelines. However, integrated findings reveal a significant **genotype–phenotype discrepancy**. While TPMT and NUDT15 reliably predict bone marrow suppression risk, they offer limited prognostic value for DILI. This evidence supports shifting from static pharmacogenetic (PGx) approaches to a dynamic, **multi-omic model** that includes secondary metabolic pathways, transporter networks, and immune-metabolic interactions.

### 4.1. Clinical and Mechanistic Implications

Phenoconversion acts as a clinical risk amplifier. Pro-inflammatory cytokines (IL-6, TNF-α, IL-1β) suppress PXR/CAR/HNF4α signaling and downregulate CYP enzymes and transporters, temporarily converting genotypic normal metabolizers into functional poor metabolizers. This reduces hepatic clearance capacity, redirects metabolic flux toward hepatotoxic pathways, such as increased 6-MMP formation or impaired MTX elimination, and increases the risk of hepatotoxicity during inflammation or infection. Recognizing phenoconversion shifts risk assessment from static, genotype-centric models to dynamic, state-aware prognostication.

Transporters play a critical role in tissue-specific drug exposure. Variants in *SLCO1B1*, *ABCC2/4*, and related genes alter the balance between hepatocyte uptake and efflux, allowing toxic metabolites to accumulate intracellularly regardless of serum levels. Inflammatory states further suppress transporter expression and localization, creating a closed hepatocyte state that increases susceptibility to cholestasis and parenchymal injury. Thus, transporter genetics and regulation often explain discrepancies between serum and liver toxicity and should be prioritized in expanded pharmacogenomic panels.

Secondary metabolic pathways and polypharmacy influence hepatotoxic risk. Reduced-function CYP alleles (*CYP2C9*, *CYP3A4/5*, *CYP2D6*) and inducible *CYP2E1* variants interact with co-medications like azoles, TMP-SMX, and NSAIDs, causing metabolic bottlenecks. These interactions increase MTX and 6-MP retention or redirect their metabolism toward hepatotoxic metabolites. Clinically relevant drug–drug interaction scenarios, called the triple-threat, cause multiplicative risk increases beyond individual genetic variant effects.

Integrating multi-omics data enhances actionable sensitivity for risk prediction by linking extended PGx, including primary and secondary genes, with longitudinal transcriptomics, targeted metabolomics (6-TGN, 6-MMP, MTX/7-OH-MTX, GSH), and cytokine profiling (CRP, IL-6, TNF-α). This dataset enables earlier, mechanistically informed detection of phenoconversion, identification of toxic metabolic shunts, and the establishment of empirically derived thresholds for dose modification that surpass those of genotype-only stratification. In clinical practice, combining PGx with serial molecular and biochemical markers, such as liver enzymes, metabolite panels, and inflammatory cytokines, forms a practical monitoring bundle. This bundle identifies patients at imminent risk and facilitates preemptive interventions. When integrated into electronic medical record (EMR)-based clinical decision support systems, these signals can drive automated dosing recommendations, alert clinicians to increasing ecological risk (eco-score), and prioritize resource-intensive interventions for those most likely to benefit. Consequently, a multimodal strategy enables real-time therapeutic recalibration, reduces severe hepatotoxic events and unnecessary treatment interruptions, and improves drug utilization and cost-effectiveness. This delivers measurable ecological and clinical value when selectively implemented within routine care pathways.

### 4.2. The Expanded Genetic Test and Future Strategies

The rationale for expanding the PGx panel is supported by emerging empirical evidence. Adding secondary pharmacogenes such as *ITPA*, *PNPLA3*, and *MTHFR*, along with transporter variants such as *ABCC2* and *SLCO1B1*, to routine *TPMT/NUDT15* genotyping may increase the number of patients identified as having a mechanistic risk for 6-MP/MTX hepatotoxicity. However, this approach requires further validation through rigorous qualification studies. The potential benefit is clearest when genotype data are interpreted within a validated, dynamic care pathway. While genotype establishes initial risk categories, cytokine profiling, and therapeutic drug monitoring (TDM) provide additional temporal signals to detect phenoconversion and actionable metabolic changes. To effectively support clinical practice, the expanded panel should be integrated into: (1) prespecified decision thresholds, such as genotype and biomarker composite scores, prospectively shown to predict predefined outcomes (for example, grade ≥ 3 hepatotoxicity or treatment interruption) with sufficient sensitivity, specificity, positive predictive value, and net reclassification improvement over standard care; (2) EMR-integrated clinical decision support (CDS) systems that translate composite scores into explicit dosing and monitoring recommendations; and (3) a validated workflow for confirmatory functional assessments, such as probe-substrate assays or pharmacokinetic (PK) sampling, when biomarker signals reach action thresholds.

An expanded panel may be a promising strategy to improve prevention of 6-MP/MTX hepatic toxicity, provided its implementation is guided by prospective evidence showing that genotype-plus-dynamic-biomarker algorithms deliver reproducible, actionable risk stratification, support better patient outcomes, and remain sustainable in routine clinical practice.

## 5. Conclusions

The pharmacological synergy between 6-MP and MTX remains the cornerstone of maintenance therapy in ALL. However, the clinical safety of this regimen does not meet the stringent requirements of precision medicine. This review shows that although conventional **TPMT** and **NUDT15** genotyping is essential and effective for reducing myelosuppression, it is inadequate for addressing the **genotype–phenotype discrepancy** in hepatotoxicity. *TPMT* and *NUDT15* genotyping effectively reduces myelosuppression risk but does not address the genotype–phenotype gap in hepatotoxicity. Integrated analyses show that DILI during 6-MP/MTX co-therapy results from dynamic network dysfunction. This dysfunction is driven by cytokine-mediated phenoconversion, inflammation-induced downregulation of hepatic transporters such as SLCO1B1 and ABCC2/4, and metabolic consequences of polypharmacy. These factors cause toxic metabolite accumulation that single-gene analysis cannot predict. Therefore, a comprehensive therapeutic safety assessment should include both static genomic information and dynamic physiological parameters, especially inflammatory status and drug–drug interactions.

Advancing the field requires routine monitoring of inflammatory markers, such as CRP and IL-6, in risk assessment protocols for 6-MP/MTX maintenance therapy. Piloting multidisciplinary alert systems to identify high-risk patients based on cytokine profiles and polypharmacy is also recommended. These approaches should undergo prospective validation to assess their effectiveness in reducing DILI incidence and improving clinical outcomes. Establishing collaborative registries with standardized DILI phenotyping, biobanking, and shared modeling platforms will support robust replication and refinement of predictive algorithms. Ultimately, adaptive integration of multi-omics data into clinical workflows may bridge the genotype–phenotype gap, reduce hepatotoxicity, and provide a model for precision management in complex, multi-agent oncology regimens.

## Figures and Tables

**Table 2:** Conceptual hepatotoxicity risk scoring of key pharmacogenetic constellations during 6-MP and MTX co-therapy.

Gene Constellation	CHRS (1–10)	Risk Level	Pharmacological Interpretation and Clinical Rationale
Wild-type panel (*TPMT*, *NUDT15*, *SLCO1B1*, *ABCC2*, *PNPLA3*, *MTHFR*, *CYP2C9/3A4/2D6* WT)	1–2	Minimal	Intact primary detoxification (TPMT/NUDT15), transport (SLCO1B1/ABCC2), folate and lipid metabolism (MTHFR/PNPLA3), and **CYP-mediated clearance ensure a low baseline DILI risk** under guideline-concordant 6-MP + MTX dosing.
Single primary defect TPMT *3A/*3A hom or *NUDT15*	7–8	High	Severe impairment of methylation or nucleotide detoxification causes excessive 6-TGN buildup, disrupts the 6-TGN/6-TMMP balance, and significantly increases vulnerability to acute thiopurine-induced liver injury and systemic toxicity. Requires a major dose reduction and close therapeutic drug monitoring.
Combined primary defects *TPMT* hom + *NUDT15* hom	9–10	Critical	Concurrent loss of methylation and NUDT15-mediated dephosphorylation virtually eliminates primary detoxification, resulting in extreme intracellular 6-TGN buildup, significant metabolic stress, and a critical risk of fulminant DILI. Thiopurine use is generally contraindicated or restricted to very low doses with intensive monitoring.
Primary defect + hepatic transporters e.g., *TPMT* or *NUDT15* hom + *SLCO1B1* hom and/or *ABCC2* hom	8–9	Very High	Impaired thiopurine detoxification combined with reduced hepatic uptake (SLCO1B1) and/or impaired biliary efflux (ABCC2) promotes intrahepatic trapping of 6-MP/MTX conjugates and bile acids, significantly increasing the risk of cholestatic or mixed-pattern DILI during combination therapy.
Primary defect + *PNPLA3* hom	8–9	Very High	Thiopurine and MTX-induced stress are superimposed on a genetic predisposition to steatosis and fibrosis, reducing the liver’s capacity to regenerate. This combination shifts the risk from isolated acute parenchymal injury toward faster chronic progression (steatohepatitis, fibrosis).
*NUDT15* hom + *MTHFR* hom	8–9	Very High	Combined disruption of nucleotide metabolism (NUDT15) and folate-dependent pathways (MTHFR) worsens mitochondrial and metabolic stress under 6-MP + MTX. Reduced MTX clearance and altered folate leve**ls** together increase the risk of liver and mitochondrial damage.
Primary defect + reduced-function CYPs e.g., *TPMT* or *NUDT15* hom + CYP2C9*2/*3 ± *CYP3A4/3A5*, *CYP2D6*	7–8	High/Very High	Impaired thiopurine detoxification, combined with a reduction in CYP-mediated clearance of co-medications (NSAIDs, azoles, SSRIs, etc.), increases oxidative and mitochondrial stress. Pharmacological phenoconversion caused by CYP deficiency indirectly worsens thiopurine-induced DILI during polypharmacy.
Extended constellation primary defects + *SLCO1B1/ABCC2* + *PNPLA3* + reduced-function CYPs	10	Critical	Convergent impairment of primary detoxification, hepatic transport, regenerative capacity, and CYP-mediated co-medication clearance results in maximum cumulative hepatic stress. This combination indicates an extremely high-risk group for acute DILI, cholestasis, and rapid progression to chronic liver disease; use of 6-MP/MTX should be highly individualized, with careful consideration of alternative regimens.

**Table 3:** Clinical Decision Support Matrix: Integrated PGx and Dynamic Biomarker-Based Monitoring.

Category	Standard Risk (Wild-Type Profile)	High Risk
Genetic Background (Extended PGx)	Negative for variants in *TPMT*, *NUDT15*, *ITPA*, *MTHFR*, *ABCC2*, *SLCO1B1*, and *CYP2C9* genes.	Presence of actionable variants (e.g., PM or non-functional alleles) on the panel.
Clinical Phenotype	Stable baseline disease, normal renal function, no significant inflammation.	ALL, IBD with polypharmacy, decreased GFR, or chronic inflammatory state.
Initial Dosing Strategy	Standard starting dose according to current professional clinical guidelines.	Preemptive dose reduction based on the severity of genetic and transporter risk.
Dynamic Biomarkers	Periodic measurement of ALT, AST, Bilirubin, and GFR.	Intensive monitoring: ALT, AST, Bilirubin, GGT, 6-TGN, 6-MMP, MTX-PG + cytokines (IL-6, TNF-α) + CRP
Monitoring Frequency	Standard follow-up (e.g., every 4–8 weeks).	Strict surveillance: Frequent sampling for early detection of phenoconversion and hepatic stress.
Therapeutic Response Options	Routine follow-up, dose escalation based on efficacy.	Model-based dose modification or brief therapy interruption to avoid transporter blockade and toxicity.
Clinical Objective	Maintenance of the therapeutic window.	Preventing Grade 3+ hepatotoxicity and ensuring treatment continuity.

## Data Availability

The data presented in this study are available within the article (see Table 1 and Table 2, and Figure 1). The risk estimates and synergistic models were synthesized from publicly available datasets and clinical studies cited in the references.

## Abbreviations

The following abbreviations are used in this manuscript:

ALT	Alanine Aminotransferase
AST	Aspartate Aminotransferase
6-MMP	6-methylmercaptopurine
6-MP	6-mercaptopurine
6-MP/MTX co-therapy	Combined 6-mercaptopurine and methotrexate therapy
6-TGN	6-thioguanine nucleotides
6-TGTP	6-thioguanine triphosphate
ABCB1	ATP-binding cassette sub-family B member 1 (P-glycoprotein)
ABCC2	ATP-binding cassette sub-family C member 2 (MRP2)
ABCC4	ATP-binding cassette sub-family C member 4 (MRP4)
AI/CRP	C-reactive protein (CRP)
AOX	Aldehyde oxidase
CAR	Constitutive androstane receptor (NR1I3)
CPIC	Clinical Pharmacogenetics Implementation Consortium
CYP	Cytochrome P450 enzyme family
CYP2C9	Cytochrome P450 2C9
CYP2D6	Cytochrome P450 2D6
CYP2E1	Cytochrome P450 2E1
CYP3A4	Cytochrome P450 3A4
DILI	Drug-induced liver injury
DDI	Drug–drug interaction
DHFR	Dihydrofolate reductase
DPWG	Dutch Pharmacogenetics Working Group
GSH	Glutathione
HNF4α	Hepatocyte nuclear factor 4 alpha
IBD	Inflammatory bowel disease
IL-1β	Interleukin-1 beta
IL-6	Interleukin-6
ITPA	Inosine triphosphate pyrophosphatase
MRP2	Multidrug resistance-associated protein 2 (ABCC2)
MRP4	Multidrug resistance-associated protein 4 (ABCC4)
MTX	Methotrexate
MTX-PG(s)	Methotrexate polyglutamates
NF-κB	Nuclear factor kappa-light-chain-enhancer of activated B cells
NUDT15	Nudix hydrolase 15
OATP1B1	Organic anion-transporting polypeptide 1B1 (SLCO1B1)
OR	Odds ratio
PXR	Pregnane X receptor (NR1I2)
PGx	Pharmacogenetics/Pharmacogenomics
PNPLA3	Patatin-like phospholipase domain containing 3
PR/STAT3	Signal transducer and activator of transcription 3 (STAT3)/JAK–STAT pathway (JAK implied)
RXR	Retinoid X receptor
SLC19A1	Solute carrier family 19 member 1 (reduced folate carrier)
SLCO1B1	Solute carrier organic anion transporter family member 1B1 (OATP1B1)
TMP-SMX	Trimethoprim–sulfamethoxazole
TNF-α	Tumor necrosis factor alpha
TDM	Therapeutic drug monitoring
TM6SF2	Transmembrane 6 superfamily member 2
TPMT	Thiopurine S-methyltransferase
TGFB1	Transforming growth factor beta 1
